# Effects of poly(3-hydroxybutyrate) [P(3HB)] coating on the bacterial communities of artificial structures

**DOI:** 10.1371/journal.pone.0300929

**Published:** 2024-04-18

**Authors:** Yee Jean Chai, Taufiq Ahmad Syauqi, Kumar Sudesh, Tan Leng Ee, Cheah Chee Ban, Amanda Chong Kar Mun, Elisabeth Marijke Anne Strain, Faradina Merican, Masazurah A. Rahim, Kaharudin Md Salleh, Chee Su Yin

**Affiliations:** 1 Centre for Global Sustainability Studies, Universiti Sains Malaysia, Minden, Penang, Malaysia; 2 School of Biological Sciences, Universiti Sains Malaysia, Minden, Penang, Malaysia; 3 School of Housing, Building and Planning, Universiti Sains Malaysia, Minden, Penang, Malaysia; 4 Institute for Marine and Antarctic Studies, University of Tasmania, Hobart, Australia; 5 Centre for Marine Socioecology, University of Tasmania, Hobart, Australia; 6 Fisheries Research Institute, Batu Maung, Penang, Malaysia; Konkuk University, REPUBLIC OF KOREA

## Abstract

The expanding urbanization of coastal areas has led to increased ocean sprawl, which has had both physical and chemical adverse effects on marine and coastal ecosystems. To maintain the health and functionality of these ecosystems, it is imperative to develop effective solutions. One such solution involves the use of biodegradable polymers as bioactive coatings to enhance the bioreceptivity of marine and coastal infrastructures. Our study aimed to explore two main objectives: (1) investigate PHA-degrading bacteria on polymer-coated surfaces and in surrounding seawater, and (2) comparing biofilm colonization between surfaces with and without the polymer coating. We applied poly(3-hydroxybutyrate) [P(3HB)) coatings on concrete surfaces at concentrations of 1% and 6% w/v, with varying numbers of coating cycles (1, 3, and 6). Our findings revealed that the addition of P(3HB) indeed promoted accelerated biofilm growth on the coated surfaces, resulting in an occupied area approximately 50% to 100% larger than that observed in the negative control. This indicates a remarkable enhancement, with the biofilm expanding at a rate roughly 1.5 to 2 times faster than the untreated surfaces. We observed noteworthy distinctions in biofilm growth patterns based on varying concentration and number of coating cycles. Interestingly, treatments with low concentration and high coating cycles exhibited comparable biofilm enhancements to those with high concentrations and low coating cycles. Further investigation into the bacterial communities responsible for the degradation of P(3HB) coatings identified mostly common and widespread strains but found no relation between the concentration and coating cycles. Nevertheless, this microbial degradation process was found to be highly efficient, manifesting noticeable effects within a single month. While these initial findings are promising, it’s essential to conduct tests under natural conditions to validate the applicability of this approach. Nonetheless, our study represents a novel and bio-based ecological engineering strategy for enhancing the bioreceptivity of marine and coastal structures.

## Introduction

By 2025, more than half the global population will reside within 200 km of coastlines [1]. This rapid urbanization has spurred “ocean sprawl”, expanding human activity and infrastructure into coastal and marine areas [2,3]. Coastal structures like ports and harbours are vital for trade, transportation, and economic growth in these regions [4,5]. Concrete serves as the primary material for constructing these infrastructures due to its durability, strength, and adaptability [6,7]. However, extensive concrete use in construction poses significant environmental challenges for marine ecosystems [8,9]. Concrete structures displace natural coastal habitats, altering vital nursery environments like coral reefs, seagrass beds and mangroves [10,11]. Additionally, the release of alkaline substances from concrete can elevate pH levels in surrounding waters, impacting pH-sensitive marine organisms [12].

In the pursuit of curbing ocean sprawl and fostering sustainable urban development, researchers and engineers are exploring innovative strategies to improve concrete-based strictures for ecosystem restoration [3,13]. While previous eco-engineering approaches prioritized structural complexity [14,15], the focus has shifted toward investigating various material types [16,17]. Despite numerous studies examining different materials for artificial structures, conclusive evidence on their significant impact on macrofaunal species richness, community compositions, and related indicators remains elusive [18–20]. One innovative approach involves enhancing artificial structure bioreceptivity through surface treatment [21]. This technique aims to modify surface attributes to attract and facilitate the growth of marine organisms [22–24]. By creating a conducive environment for marine organisms, surface treatment encourages the establishment of diverse ecosystems, contributing to coastal environment restoration and conservation.

In recent years, substantial research has probed into the influence of surface coatings composed of various compounds on biofilm formation. However, the majority of these studies have primarily concentrated on evaluating the antifouling effects rather than accentuating the growth of marine biofilms or microorganisms within the marine ecosystem [25–27]. Despite this, a niche area of study has emerged concentrating on enhancing biofilms, particularly steering attention towards bio-based compounds. An intriguing exploration has revolved around utilizing extracted algal extracellular polymeric substances as surface coatings [28,29], demonstrating significant efficacy in enhancing biofilm recruitment by up to 231% [30]. Additionally, research exploring chitosan-coated lignin nanoparticles has surfaced as another pathway for boosting biofilm growth. Lignin, a natural polymer derived from plant cell, and chitosan, sourced from crustacean shells, have shown promising effects in stimulating a 25–45% higher microbial attachment and growth rate [31]. These examples underscore the diverse range of bio-based compounds with the potential to enhance biofilm recruitment and bolster the ecological balance within marine environments, highlighting the outcomes of prior studies. This success has steered the focus towards exploring other biodegradable materials, particularly directing attention to polyhydroxyalkanoates (PHAs). This shift signifies a continuation from the success observed in previous biofilm enhancement studies, encouraging a deeper investigation into PHAs as sustainable alternatives to fortify marine biofilm growth and support ecological sustainability.

Polyhydroxyalkanoates (PHAs) have been long studied due to their natural origin and potentially sustainable manufacturing methods [32]. These biodegradable aliphatic polyesters, synthesized by various bacteria using renewable resources [33], serve as eco-friendly alternatives to non-degradable plastics like polyethylene and polypropylene [34]. With over a hundred reported variants, common PHA types include poly(3-hydroxybutyrate) [P(3HB)], poly(3-hydroxyvalerate), poly(3-hydroxybutyrate-co-4-hydroxybutyrate), and PHBHHx copolymers [35]. Their notable biodegradability in marine environments is influenced by a combination of interrelated factors, including biological, environmental, and morphological aspects [36,37], with primary biodegradation pathway occurring through enzyme-catalyzed hydrolysis [38]. In marine settings, a diverse range of microbes possess the capability to secrete extracellular PHA depolymerase enzymes, indicating their potential role in enzymatically degrading PHA in oceanic environments [37]. These PHA-degrading bacteria encompass a spectrum of approximately 24 distinct strains found within the Proteobacteria, Firmicutes, and Actinobacteria phyla, contributing significantly to the high degradability of PHAs in marine ecosystems [37,39]. The variability in the biodegradation of PHAs between marine and freshwater environments highlights the contrasting rates of degradation observed in these different settings. Studies have revealed that in marine or related conditions, PHAs undergo a notable reduction in mass of up to 31% over a span of 270 days [36]. This indicates a considerably swifter degradation process when compared to their degradation in freshwater environments, where it might take up to a year to achieve a similar reduction in mass [40,41]. This stark contrast in degradation rates underlines the distinct environmental factors and microbial activities present in these respective ecosystems that influence the breakdown of PHAs. In contrast, synthetic biodegradable polymers such as polylactic acid and polybutylene succinate exhibit considerably lower biodegradability when exposed to marine settings, showcasing a significant disparity in the degradation behaviours of various biodegradable materials in different environmental contexts [42,43].

The enzymatic degradation of PHAs in marine environments generates degradation products that serve as essential nutrients for variety of marine organisms. These breakdown by-products, serving as sources of both carbon and energy, facilitate the growth and metabolic processes of marine life [44]. The microbial degradation and mineralization processes transform these PHA degradation products into elemental components, including carbon dioxide, water, and other inorganic compounds. This conversion plays a crucial role in the cyclic recycling of carbon and nutrients in marine ecosystems, fuelling the nutrient uptake and proliferation of diverse marine bacterial communities [45]. This robust bacterial community plays a crucial role in the complex web of marine ecosystems by actively participating in the distribution of nutrients, contributing to the resilience and overall health of these delicate environments [46–48].

In ecological engineering, the utilization of biodegradable P(3HB) as surface treatments for artificial structures offers promising opportunities. The intrinsic biodegradability of P(3HB), coupled with its capacity to function as a nutritional resource for marine life, positions it as a key contender for enhancing the bioreceptivity of coastal and marine infrastructure. The delicate interplay between material degradation and marine ecology lays the foundation for a symbiotic relationship, where artificial structures become not just passive entities in the marine environment but active contributors to its vitality. The objective of our study was twofold: (1) to investigate the presence of PHA-degrading bacteria on coated surfaces and in the surrounding seawater, and (2) to compare biofilm colonization patterns between surfaces with and without the PHA coating. By exploring these factors, we sought to understand the potential of this biodegradable coating to enhance bioreceptivity and facilitate ecological connectivity in urban coastal environments.

## Materials and methods

### Preparation of concrete samples

Two types of concrete cubes were prepared: green concrete with a polymer coating and Portland cement concrete without any coating. The green concrete was formulated by incorporating ground granulated blast-furnace slag (GGBS), quarry dust, and seashells as partial replacements for standard CEM-1 cement and river sand. On the other hand, the Portland cement concrete was made using the CEM-1 cement and river sand without any additives or replacements. The specific mix proportions were provided in S1 Table.

To ensure ease of demoulding without the need for a release agent, all concrete sample were cast in square-shaped silicone moulds measuring 20 mm x 20 mm x 20 mm. After a period of 24 hours, the sample was demoulded and allowed to undergo air-curing at room temperature, maintained at 26–28°C, for a duration of 7 days prior to coating process.

### P(3HB) extraction and purification

In the pursuit of identifying an optimal biopolymer for surface coating applications, the selection of P(3HB) is grounded in its distinct advantages within the context of marine environments. Its selection is particularly driven by its notable attributes, including high biodegradability in marine settings, favourable biocompatibility, well established production availability, and suitability for handling procedures. The P(3HB) [molecular weight (Mw): 6.3 × 104; polydispersity (Mw/Mn): 2.05], used in this study, was biosynthesized using *Cupriavidus necator* H16 with palm olein as a carbon source [49]. The lyophilized cells were fed to yellow mealworms (the larval phase of a mealworm beetle, *Tenebrio molitor*), where the bacterial cells were digested. The indigestible P(3HB) was subsequently excreted by the mealworms in the form of faecal pellets. After the biological recovery process, the P(3HB) was extracted from the faecal pellets and purified using distilled water and NaOH [50,51].

### P(3HB) coating process

**Fig 1:**
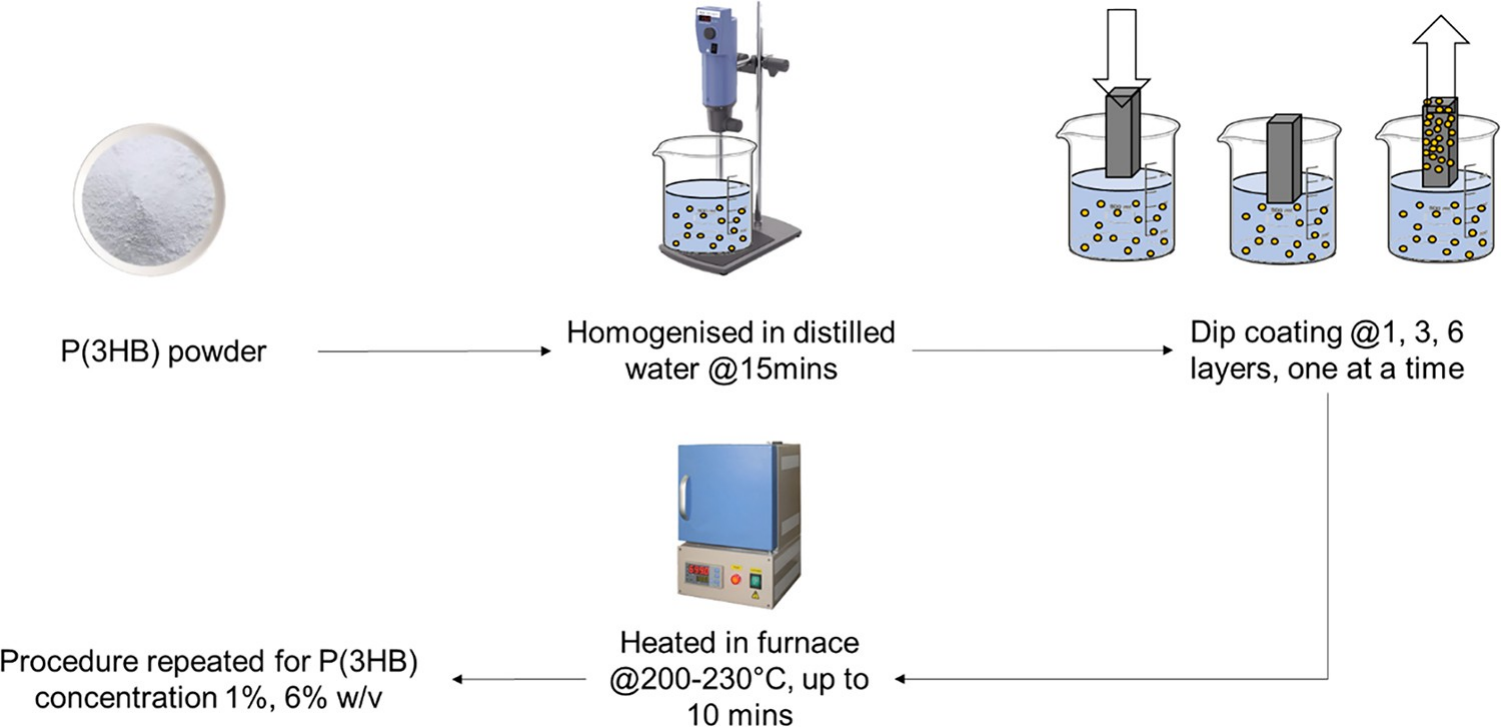
Schematic diagram on P(3HB) coating procedure.

To ensure consistent adhesion of P(3HB) particles onto the concrete sample surface, the P(3HB) powder was dispersed in distilled water for 15 minutes using a homogenizer (IKA ULTRA-TURRAX). The green concrete cubes were then coated with P(3HB) using a dip coating method (Fig 1). This involved immersing the concrete into the colloidal dispersion of P(3HB) to ensure full coverage and then slowly withdrawn to remove any excess dispersion. Subsequently, the coated concrete was transferred to a preheated furnace set to a temperature range of 200°C-230°C and held for 10 minutes. This thermal treatment step facilitated the adhesion and fixation of the P(3HB) coating onto the surface of the concrete sample. For higher numbers of coating cycles, the coated concrete sample was subjected to air-cooling, followed by subsequent dipping and heating in the furnace. This iterative process of dipping, heating, and air-cooling was repeated until the desired number of coating cycles was achieved.

To generate concrete samples with various concentrations and coating thicknesses, we conducted numerous iterations of the dip-coating, furnace drying, and air-cooling procedures while modulating the concentration of P(3HB) in the colloidal dispersion. The specific details of the treatment, including the P(3HB) concentration and the number of coating cycles, can be found in Table 1.

**Table 1:** The experimental setup included three replicate samples for each treatment, with an additional two concrete samples serving as experimental controls.

Treatment	P(3HB) concentration, % w/v	P(3HB) coating cycles
Portland cement concrete (Negative control), CO	0%	0 time
Green concrete (Positive control), GO	0%	0 time
G-11	1%	1 time
G-13	1%	3 times
G-16	1%	6 times
G-61	6%	1 time
G-63	6%	3 times
G-66	6%	6 times

### P(3HB) quantification on concrete surface

The surface morphology of the P(3HB) coating layer on the concrete surface was assessed through scanning electron microscopy (SEM). The examination was carried out under conditions of 20 Pa pressure and a 15 kV acceleration voltage to prevent sample charging. To investigate the composition and structure, an energy-dispersive X-ray spectroscopy (EDX) detector was utilized. Moreover, the structure of the extracted P(3HB) powder underwent analysis. Fourier-transform infrared spectroscopy (FTIR) was employed to examine the functional groups present in the P(3HB) layers. Spectral scans were conducted within the range of 4000 to 400 cm^-1^, maintaining a resolution of 4 cm^-1^. Subsequently, the impact of escalating P(3HB) coating cycles on the deposited carbon quantity (n = 6) was assessed through a one-way ANOVA using R (version 4.1.1). It is crucial to acknowledge that the statistical power and robustness of findings may be constrained by the small sample size in the experimental setting of the current study. Therefore, for each statistical analysis conducted, diagnostic tools such as residual vs. fitted plots and normal QQ plots were systematically examined to ensure the validity and reliability of the employed ANOVA. Experimental setup for biofilm colonization.

**Fig 2:**
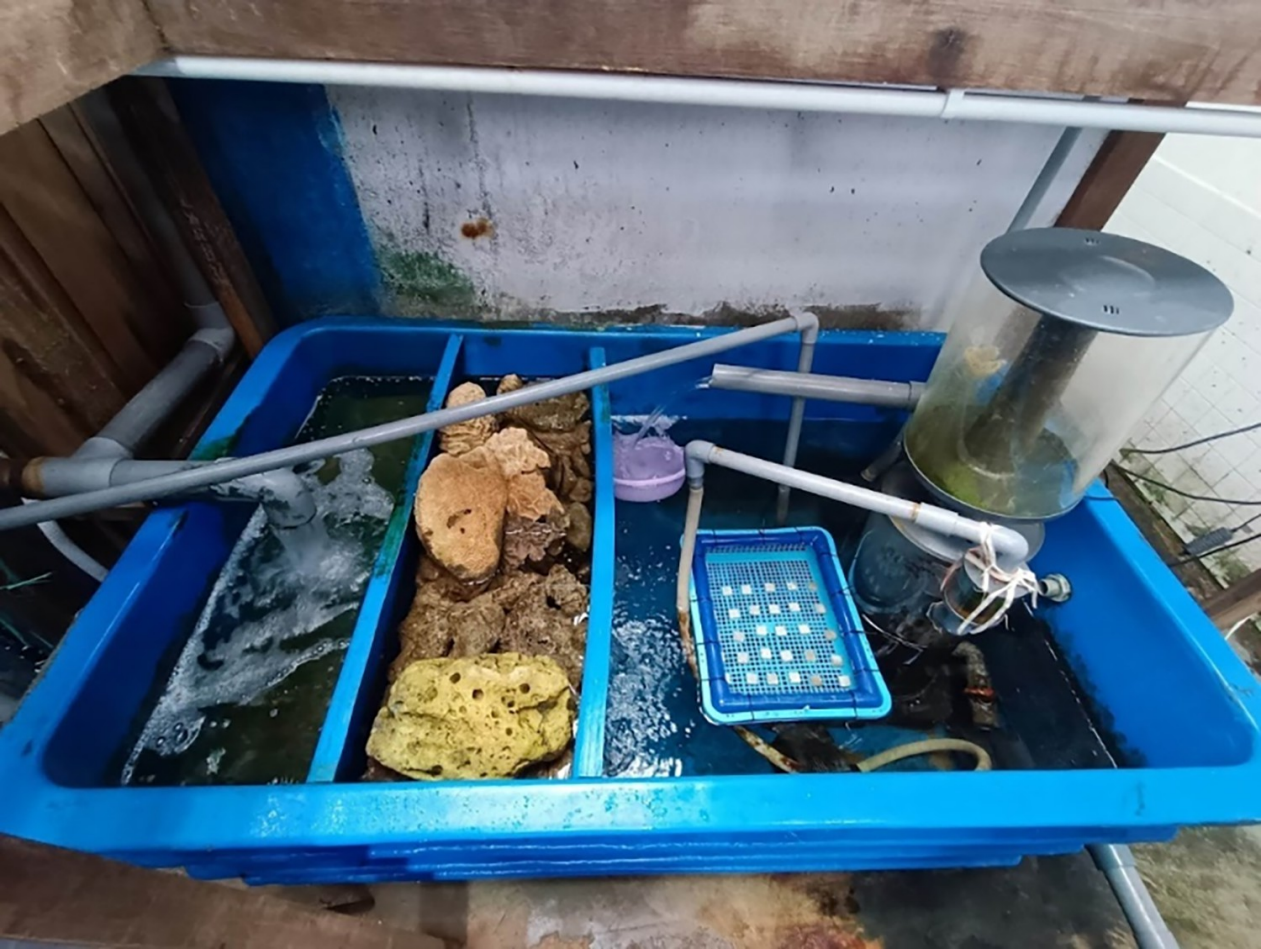
Filtration tank coupled with sand, coral and protein skimmer respectively. Samples were placed in a floating tray.

Within 48 hours of the coating process, all P(3HB) coated concrete, along with the negative and positive control concretes (CO and GO), were transferred to an experimental seawater tank. This tank was located in an indoor area with access to natural sunlight through a skylight window, simulating conditions closer to those in a natural marine environment. The filtration tank system was specifically designed with three compartments to facilitate efficient filtration and recirculation of seawater (Fig 2). To maintain separation and prevent seawater from mixing, each compartment’s water level was kept lower than the previous one. The seawater intake was sourced from the coastal area (Batu Maung, Penang, Malaysia), ensuring that its conditions consistently fell within the range: a temperature of 29 ± 0.5°C, a dissolved oxygen level of 5 ± 0.5 mg/L, a salinity of 29 ± 1.5 ppt, and a pH level of 7.7 ± 0.1.

A total of twenty-four concrete samples were carefully placed in a tray holder, ensuring they did not contact each other. The tray was positioned on the water surface to ensure complete submersion of the sample. Once positioned, the samples were left undisturbed for a period of 28 days, allowing for uninterrupted bacterial colonization to occur. To document the visual changes throughout the experimental period, we took two photographs of the entire upper surface of the concrete samples—one at the beginning and another at the end of the experimental timeline. These photographs were used to assess the extent of biofilm coverage on the surface of the concrete.

### Percentage coverage of biofilm colonization

Among the twenty-four samples, with triplicates for each of the eight treatments, the most representative photograph was chosen for every treatment. These captured images of the samples were then processed using ImageJ (version 1.8.0) to perform arithmetic and logical operations using the image calculator [52]. The images of each treatment, taken before and after the experiment, were first cropped and aligned to ensure proper alignment. Subsequently, both images were converted to 8-bit grayscale to enhance the visibility of biofilm growth on the concrete surface.

To assess the changes in biofilm growth, the bitmap images before and after the experiment were compared using the Image Calculator with the subtract operator. The subtract operation in the Image Calculator allows for a pixel-by-pixel comparison between the before and after images. By subtracting the pixel values of the before image from the after image, a resulting image is generated. This resulting image highlights the areas where changes have occurred, indicating the presence or absence of biofilm growth. A threshold value of 40% was chosen, served as a decisive marker to distinguish regions of substantial biofilm growth. Pixels surpassing this threshold signify a significant increase in biofilm coverage, whereas pixels falling below it indicate areas where little to no biofilm growth has occurred. The percentage of white pixels in this resulting image was calculated to determine the extent of biofilm growth on the concrete surface.

### Bacteria enumeration

After the 28-day submersion period, three seawater samples and all concrete samples were collected from the tank for microbial analysis. The samples were promptly transported to the laboratory on ice to preserve their integrity. The concrete samples were carefully weighed and then rinsed with sterile saline (0.85% w/v). A metal scraper was used to gently remove the biofilm from the entire, upward surface of the concrete samples. Following this, the samples were rinsed again with saline to eliminate any remaining microorganisms before being dried and reweighed.

To calculate the initial concentration of the biofilm in suspension, we measured the difference in concrete mass before and after washing, divided by the volume of sterile saline used. The resulting suspension underwent a series of serial dilutions and inoculated from 10^1^−10^6^ dilutions onto Zobell Marine agar 2216 in triplicate. The agar plates were then incubated at 30°C for 24–72 hours to allow for bacterial counting. The selection of Zobell Marine Agar 2216 underscores its well-established reputation as a specialized nutrient agar meticulously formulated to replicate the complex nutrient environment of marine ecosystems. This agar has gained extensive use in the laboratory cultivation of heterotrophic marine bacteria, encompassing a diverse array of genera such as *Alteromonas*, *Erythrobacter*, *Halomonas*, *Idiomarina*, *Marinobacter*, *Microbacterium*, *Oceanicaulis*, *Pseudoalteromonas*, *Pseudomonas*, *Ruegeria*, *Sulfitobacter*, *and Vibrio* [53,54].

To account for the weight of the biofilm in the dilutions, the colony forming unit (CFU) calculation was adjusted as follows:

$$CFU/ml=\frac{number\;of\;colonies\;counted\times dilution\;factor\;}{volume\;of\;suspension\;plated\;(ml)\times weight\;of\;biofilm\;(g)}$$

To assess the influence of P(3HB) concentration and coating cycles on bacterial abundance, a two-way ANOVA was conducted across all treatment groups, as well as seawater, negative and positive controls (n = 27). Post hoc testing using Tukey’s Honestly Significant Difference was then applied to evaluate specific differences among group means.

### Isolation, identification and analysis of P(3HB)-degrading bacteria

P(3HB)-degrading bacteria were isolated using the conventional clear zone method [55,56]. The prepared suspensions, as described in the section above, were inoculated onto the marine P(3HB) agar (modified Zobell Marine agar 2216). The agar medium consisted of: peptone (0.5 g/L), yeast extract (0.1 g/L), (19.45 g/L) NaCl, MgCl·6H_2_O (18.8 g/L), Na_2_SO_4_ (3.24 g/L), CaCl_2_ (1.8 g/L), KCl (0.55 g/L), NaHCO_3_ (0.16 g/L), KBr (0.08 g/L), H_3_BO_3_ (0.022 g/L), NH_4_NO_3_ (0.0016 g/L) and bacteriological agar (20 g/L); final pH 7.6 ± 0.2. Prior to sterilization, P(3HB) (0.1% w/v) was added to the medium as a polymer suspension [57]. The inoculated plates were incubated at 30°C for 7–10 days, with daily observations for the presence of clear zone formation. Bacteria showing the presence of clear zone formation were selected and underwent several subsequent subcultures to obtain pure bacterial colonies.

Isolated P(3HB)-degrading bacteria with distinct morphologies were identified through 16S rRNA gene sequence analysis. The polymerase chain reaction (PCR) amplification of the 16S rRNA gene was carried out using universal primers 27F (5’-AGAGTTTGATCMTGGCTCAG-3’) and 1492R (5’-TACGGYTACCTTGTTACGACTT-3’) [58] with SimpliAmp Thermal Cycler (Thermo Fisher Scientific, United States). The PCR reaction mixture included EconoTaq PLUS GREEN 2X Master Mix, 100 μM of each primer, 10 ng/L of genomic DNA template and nuclease-free water. The PCR conditions were as follows: an initial denaturation at 94°C for 5 min, followed by 30 cycles with denaturation at 94°C for 30 sec, annealing at 58°C for 30 sec, and extension at 72°C for 2 min. A final extension step was performed at 72°C for 5 min. The PCR product was purified using QIAquick® Gel Extraction kit (Qiagen, USA) and subsequently sent for sequencing.

Species identification based on the 16S rRNA was conducted by comparing the obtained nucleotide sequences with deposited sequences in the NCBI GenBank using the BLAST tool (http://www.ncbi.nlm.nih.gov/BLAST/). The search for sequences with high homology was limited to sequences from type material, excluding any uncultured and environmental sequences. A neighbour-joining phylogenetic tree was constructed using the software MEGA 11 with bootstrap value of 1000 replications [59].

To explore the impact of P(3HB) concentration and coating cycles on the recruitment of P(3HB) degraders, a two-way ANOVA was performed across all treatments, including both negative and positive controls (n = 8).

## Results

### Surface morphology of P(3HB) coating

**Fig 3:**
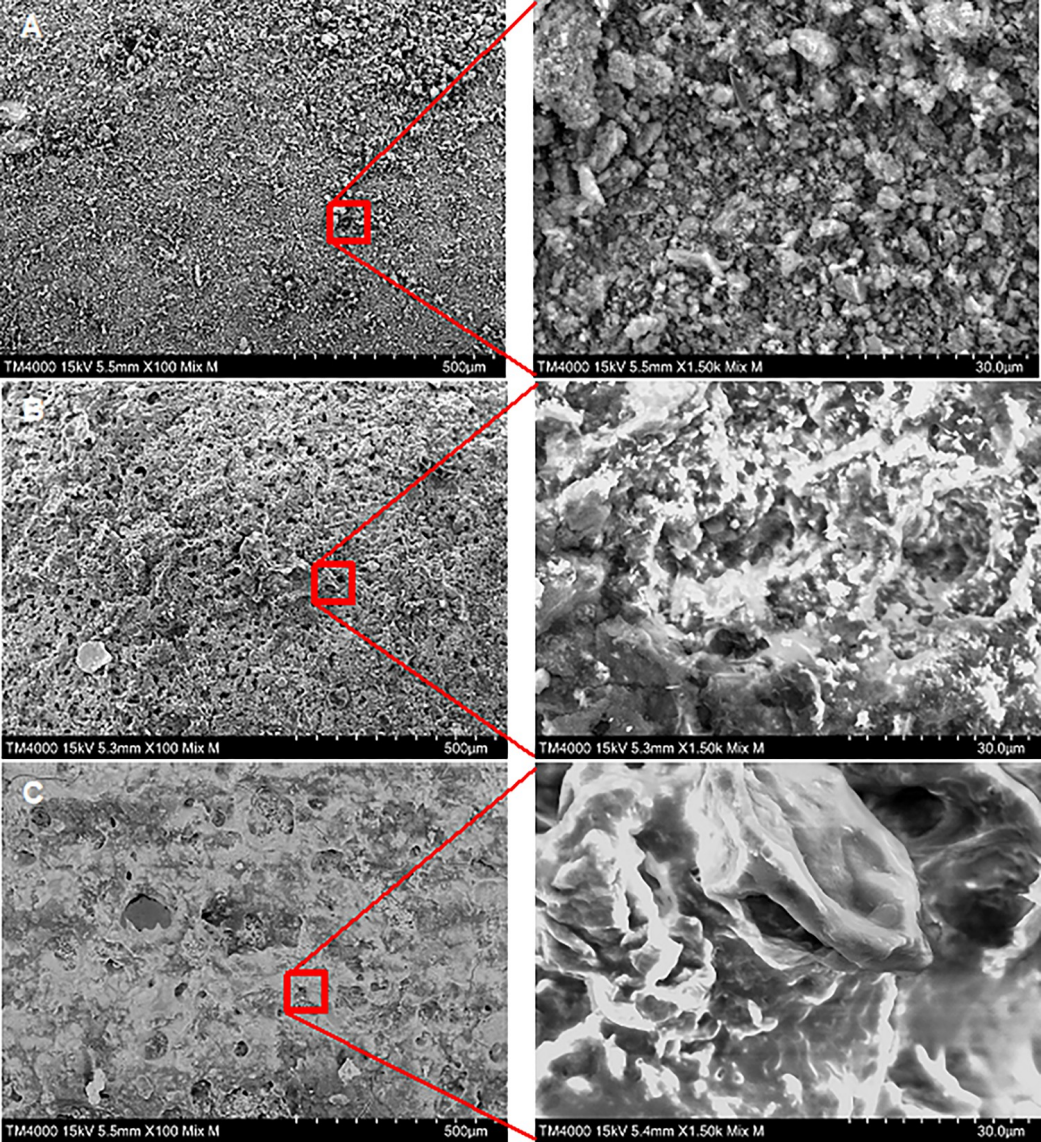
Distinctive SEM images showcasing various treatment effects on concrete surfaces. (A) Untreated green concrete surface, (B) melted 1% w/v P(3HB) and (C) melted 6% w/v P(3HB) concrete sample.

The surface of green concrete displays a unique rough texture, characterized by an irregular and uneven structure (Fig 3A). When P(3HB) was applied onto the concrete surface, a uniform and smooth layer of P(3HB) was observed, effectively covering the entire surface (Fig 3B and 3C). However, the layer left behind numerous pores or gaps in the coating. Nevertheless, a notable transformation in the surface morphology was observed when the P(3HB) concentration was increased to 6% w/v (Fig 3D). At this higher concentration, the P(3HB) coating underwent a significant change, transitioning from a porous structure to a more dense and compacted arrangement. Most of the porous features that were present at lower concentrations disappeared, resulting in a smoother, jelly-like surface.

### Chemical structure of P(3HB) coating

**Fig 4:**
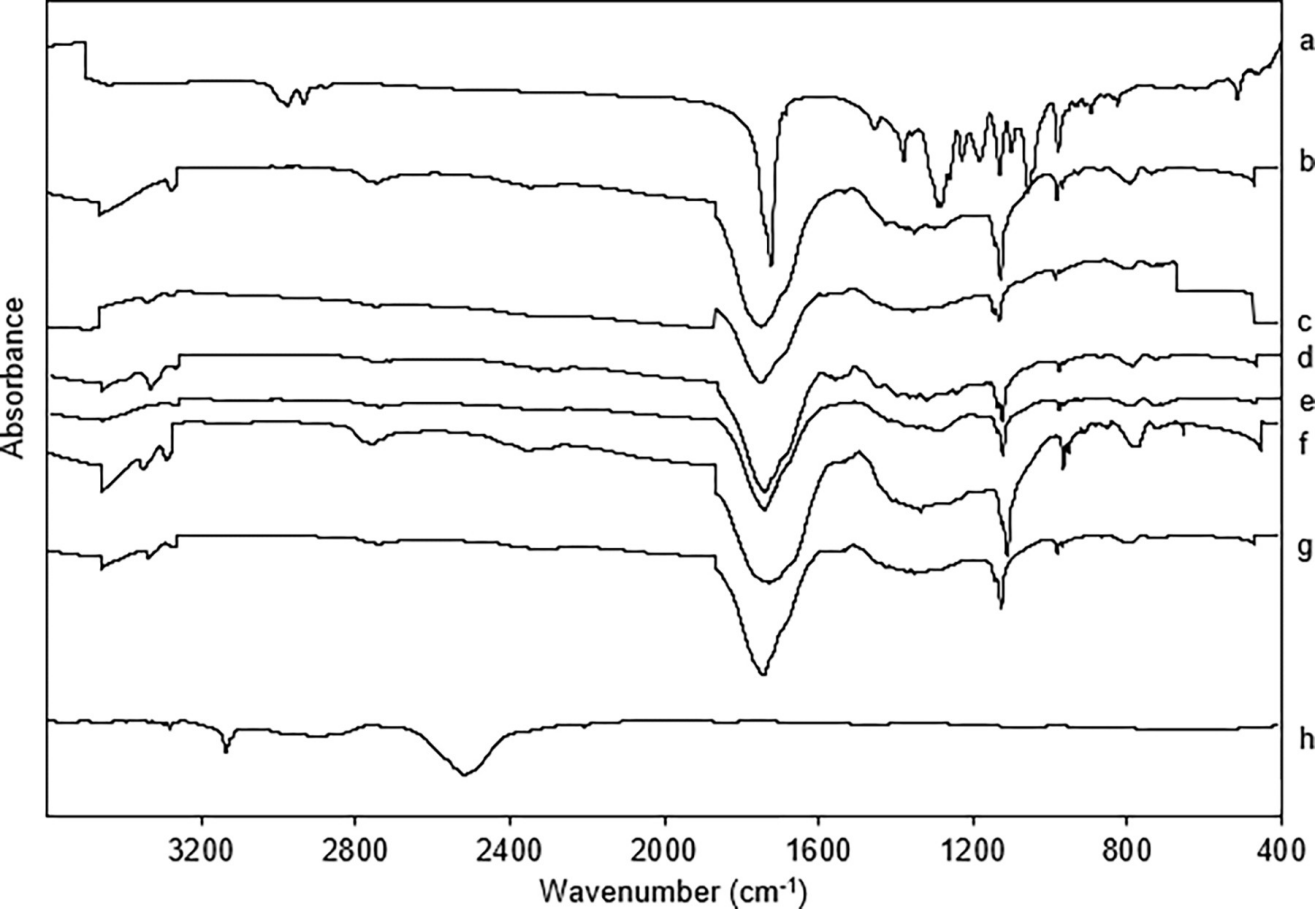
FTIR spectra analysis of concrete samples. (a) Extracted P(3HB) powder, (b) G-11, (c) G-13, (d) G-16, (e) G-61, (f) G-63, (g) G-66, and (h) green concrete control, GO.

The FTIR spectra of the P(3HB) coating layer on the concrete surface are presented in Fig 4. All treatments, except positive control without coating (GO), exhibited similar trends to that of the P(3HB) powder spectrum. The characteristic absorption peaks of P(3HB) were observed at 1720–1738 cm^-1^, indicative of the stretching vibrations of the C = O group in the ester carbonyl, and at 1125–1130 cm^-1^, which correspond to the C-O ester bond. These peaks confirm the presence of the P(3HB) coating on the concrete surface. In contrast, the spectrum of the control concrete showed peaks at 2495 cm^-1^ and 3126 cm^-1^, which are attributed to the formation of CaCO_3_, indicating that this surface was not coated with P(3HB).

EDX analysis was conducted to determine the composition of the P(3HB) coatings, revealing a hybrid organic-inorganic polymer composite. The analysis identified the predominant presence of carbon, oxygen, silicon, and calcium elements on the surface. Table 2 summarizes the relative quantities of these elements in terms of both weight and atomic percentages.

**Table 2:** EDX analysis to compare the elemental composition of the surface P(3HB) coatings at various treatment.

Element	Weight %(Atomic %)						
	Control, GO	G-11	G-13	G-16	G-61	G-63	G-66
C	7.61 (12.16)	9.77 (15.42)	45.15 (55.78)	56.37 (64.03)	34.29 (45.78)	57.87 (65.09)	56.23 (63.62)
O	55.85 (55.85)	54.77 (64.91)	41.56 (38.55)	41.04 (34.99)	44.72 (44.82)	40.74 (34.40)	42.09 (35.76)
Na	0.28 (0.23)	0.00	0.00	0.00	0.00	0.00	0.00
Mg	0.30 (0.24)	0.45 (0.35)	0.26 (0.16)	0.00	0.35 (0.23)	0.00	0.00
Al	1.20 (0.86)	1.06 (0.74)	0.58 (0.32)	0.00	0.94 (0.23)	0.00	0.00
Si	13.63 (9.31)	12.10 (8.17)	3.27 (1.73)	0.47 (0.23)	3.93 (2.24)	0.33 (0.16)	0.36 (0.17)
S	0.68 (0.41)	0.54 (0.32)	0.68 (0.31)	0.29 (0.12)	0.55 (0.27)	0.00	0.00
K	0.73 (0.36)	0.64 (0.31)	0.00	0.00	0.00	0.00	0.00
Ca	19.72 (9.44)	20.67 (9.78)	8.51 (3.15)	1.83 (0.62)	15.23 (6.09)	1.06 (0.36)	1.32 (0.45)

The EDX analysis of the green control concrete (GO) demonstrated the presence of cementitious composite compounds such as tricalcium silicate (3CaO.SiO_2_) or dicalcium silicate (2CaO.SiO_2_). This was evident from the prominent content observed for oxygen (O), silicon (Si), and calcium (Ca). In the P(3HB) coated concrete, at a low concentration of P(3HB), there was only a slight increase in the percentage of carbon atoms detected compared to the control, and similar cementitious composite compounds were detected. However, as the number of coating cycles increased while maintaining the same concentration of P(3HB), a substantial increase in the number of carbon atoms on the concrete surface was observed. Concurrently, the detection of Ca and Si atoms significantly decreased. At a high concentration of P(3HB), even a single coating cycle resulted in a higher carbon content, although significant amounts of calcium silicate were still detected. Further elevating the coating cycles at the same high concentration led to a significant increase in carbon content, though the difference was not statistically significant compared to the scenario of low concentration with a high number of coating cycles (F_2,3_ = 5.416, p = 0.101, S2 Table).

### Biofilm colonisation on concrete surface

**Fig 5:**
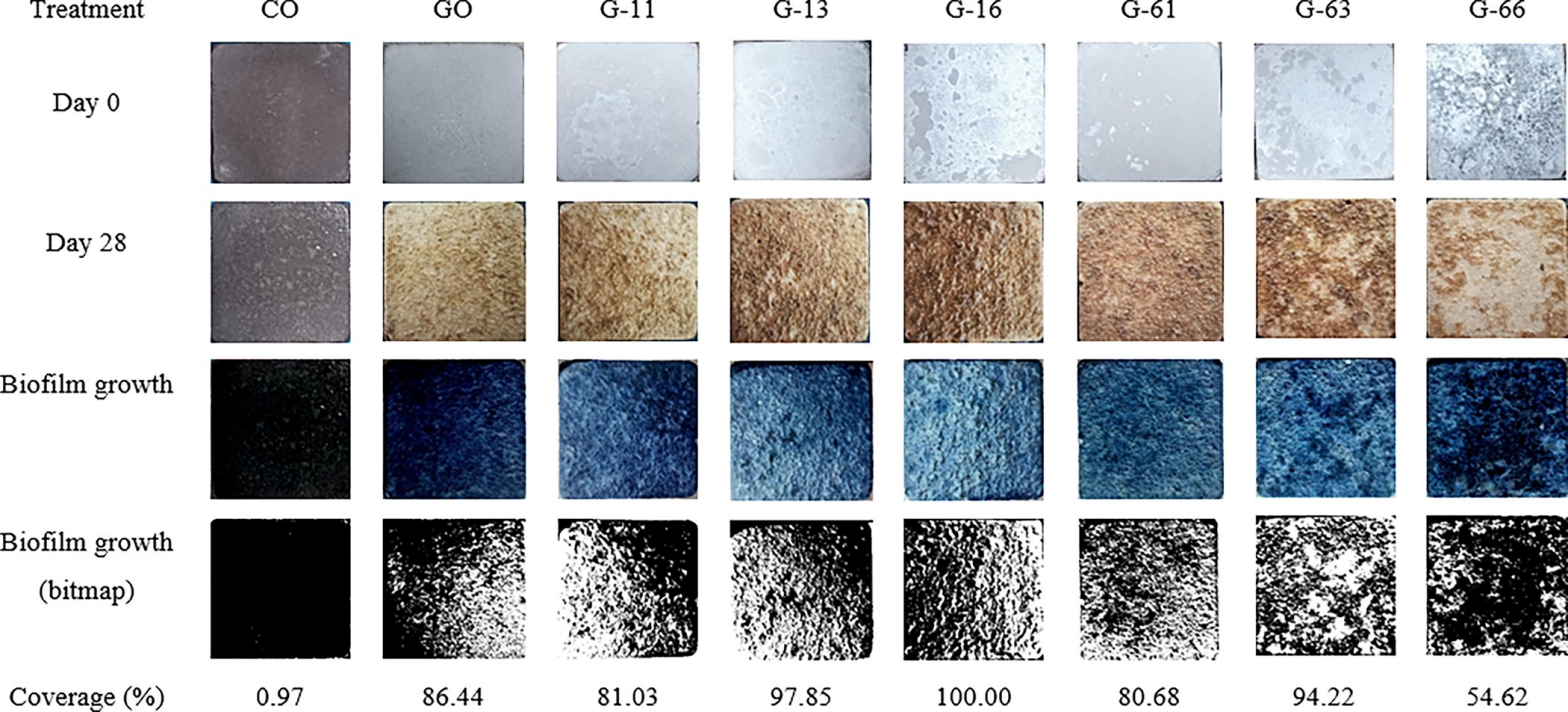
Comparative analysis of biofilm growth after 28 days of different treatments.

After 28 days of immersion in seawater, the biofilm growth patterns in the various treatment samples showed varying degrees of colonization, as shown in the Fig 5.

The negative control (Portland cement concrete, CO), displayed minimal biofilm coverage throughout the experimental period. Although some white spot decolouration was observed on the concrete surface, no recognizable living structure was apparent. In contrast, the positive control concrete, GO, demonstrated a high level of biofilm coverage. A dense brownish biofilm was visibly evident, covering a substantial portion of one edge of the concrete.

Among the P(3HB) coated concrete samples, a substantial biofilm growth exceeding 80% coverage was observed in most treatments, irrespective of the P(3HB) concentration or coating cycle used. The exception was G-66, which displayed a lower biofilm coverage of approximately 55% compared to other P(3HB) coated samples.

The low concentration and low coating cycle treatment, G-11, exhibited denser biofilm coverage on one side, similar to the positive control GO. On the other hand, treatments with low concentration and higher coating cycles (G-13 and G-16), as well as those with higher concentration and low coating cycles (G-61 and G-63), displayed more uniform biofilm coverage across the entire surface. Meanwhile, the treatment with the highest concentration and the highest coating cycle, G-66, showed biofilm occupying all edges while leaving the central part relatively uninhabited. In all cases, the coated concrete samples showcased dense and brown-reddish biofilm development, which differed from the biofilm colour observed on the positive control GO.

### Bacterial abundance on concrete surface

**Fig 6:**
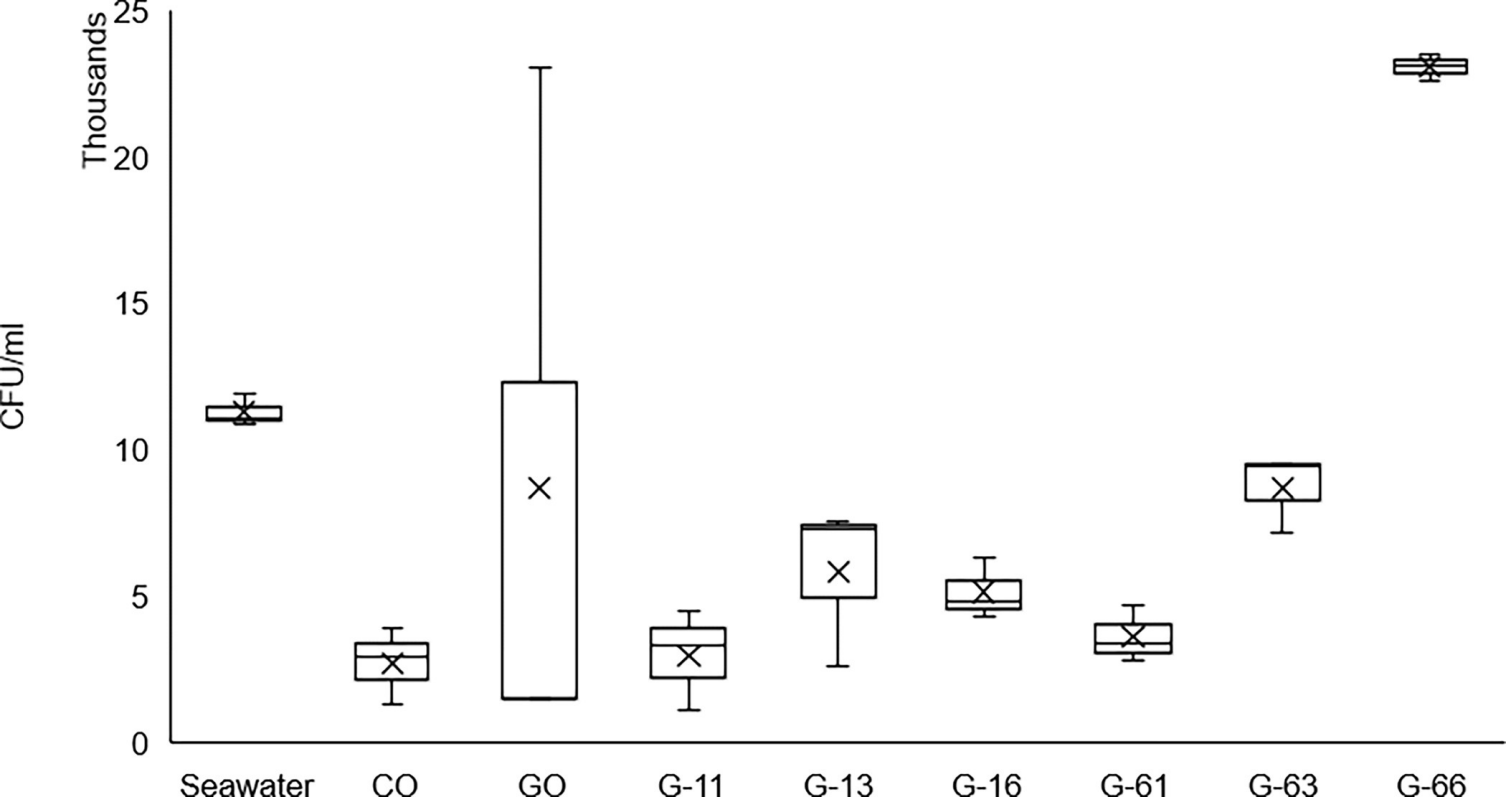
Microbial abundance of different treatment.

The impact of P(3HB) coatings on biofilm formation was further evaluated by quantifying bacterial abundance through colony-forming unit (CFU) counts. The CFU counts for the 28-day concrete samples in each treatment are presented in Fig 6. The microbial evaluation of bacteria counts on concrete surfaces revealed significant variations in bacterial abundance influenced by P(3HB) concentration (F_1,20_ = 10.080, p = 0.005), coating cycles (F_3,20_ = 5.265, p = 0.008), and their interaction (F_2,20_ = 5.790, p = 0.010, S3 Table). While the post-hoc analysis indicated a difference between using 1% and 6% P(3HB) concentration, treatments such as G-11, G-13, G-16, G-61, G-63 demonstrated a small range on the mean bacterial abundance, barring G-66 (S4 Table). A significant variance in coating cycles was observed solely between samples containing one and six cycles. Samples receiving a single coating cycle (G-11, G-61) exhibited bacterial counts similar to the negative control, CO, ranging from 4000 to 5000 CFU/ml. Nearly all treatments displayed bacterial counts approximately one magnitude lower than seawater, except GO and G-66, which exhibited higher abundances (S5 Table). Notably, significant differences in combined effects were observed between G-11 vs G-66, G-31 vs G-66, G-61 vs G-66, G-16 vs G-66, and seawater/GO/CO vs G-66. This highlights the role of G-66, involving 6% concentration and six cycles.

### Isolation and identification of P(3HB)-degrading bacteria

**Fig 7:**
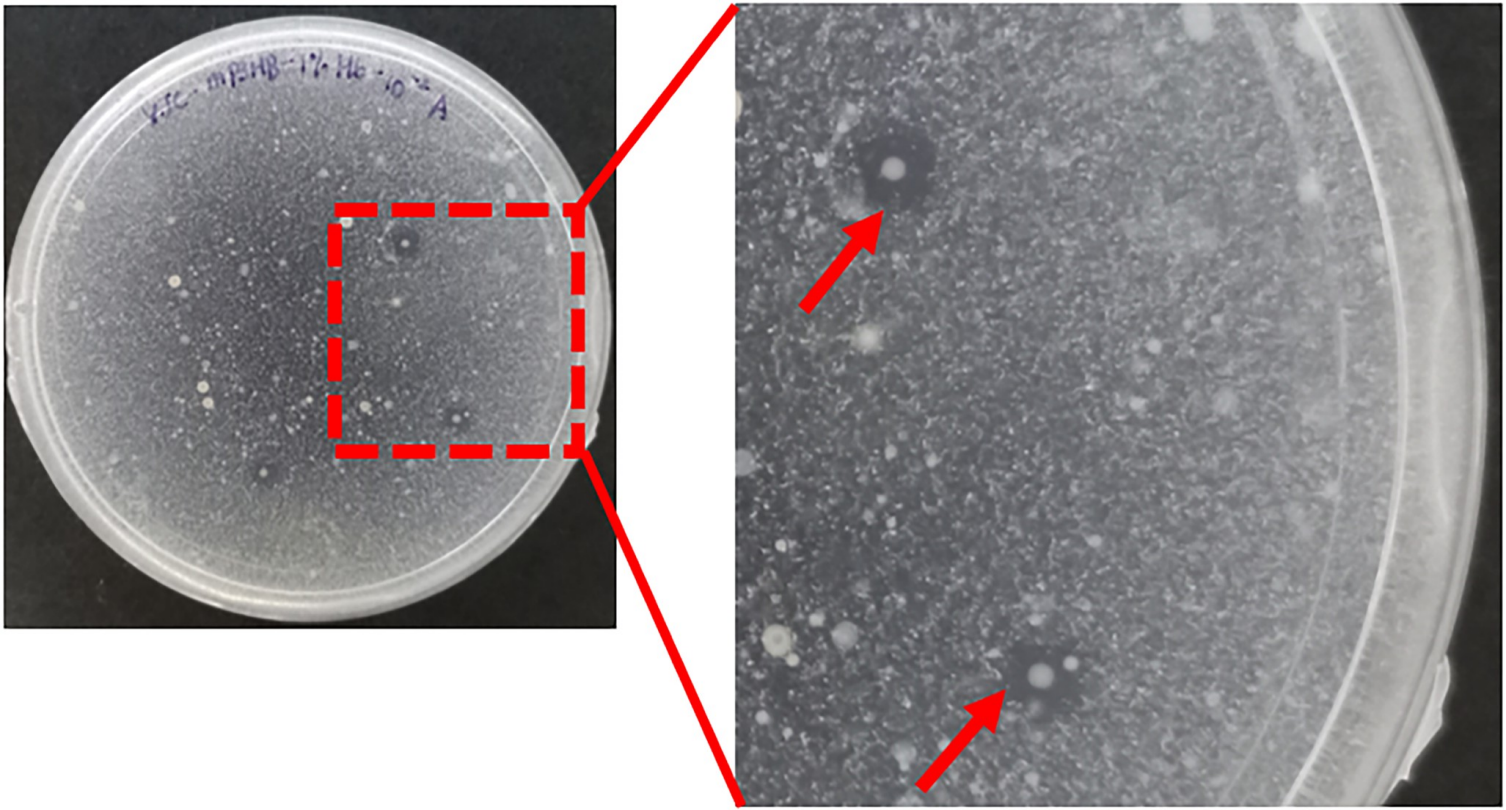
Depolymerase activity of P(3HB)-degrading bacteria as evidenced by clear zone formation.

The presence of clear zones surrounding the bacterial colonies cultured on marine P(3HB) agar indicates the activity of extracellular P(3HB) depolymerase enzymes, confirming P(3HB) degradation (Figs 7 and S1). P(3HB)-degrading bacteria were found in all treatments as evident from clear zone formation on marine P(3HB) agar, and a total of twenty-six P(3HB)-degrading bacterial strains were isolated, including seawater, negative and positive controls (Table 3). Interestingly, a higher percentage of PHA-degrading bacteria were isolated from the biofilm on concrete, irrespective of the presence of coating, P(3HB) concentration, or coating cycles, compared to seawater, except for sample G-61. No significant differences were found in the main effects of P(3HB) concentration (F_1,1_ = 0.667, p = 0.454), coating cycle (F_3,1_ = 3.125, p = 0.284), or their interactive effects (F_2,1_ = 0.500, p = 0.775, S6 Table) on the number of isolated P(3HB)-degrading bacteria. Moreover, there were no significant differences observed in the number of P(3HB)-degrading bacteria isolated from P(3HB)-coated concrete compared to both positive and negative control concretes (F_1,6_ = 0.214, p = 0.660).

**Table 3:** Number of P(3HB)-degrading bacteria strains isolated from each treatment.

Species	Seawater	CO	GO	G-11	G-13	G-16	G-61	G-63	G-66	Total
*Vibrio harveyi*	0	0	0	1	0	2	0	0	0	
*Tumebacillus lipolyticus*	0	4	1	0	2	0	0	1	1	
*Shewanella waksmanii*	1	0	0	0	0	3	0	1	0	
*Microbulbifer celer*	0	0	1	0	1	0	1	0	0	
*Marinobacter xestospongiae*	0	0	1	1	0	0	0	1	3	
Isolate	1	4	3	2	3	5	1	3	4	26

From the total of 26 isolated PHA-degrading bacteria, five strains with distinct morphological characteristics were chosen for 16S rRNA gene sequence analysis, and their gene sequences were compared with those deposited in the GenBank database. The identified P(3HB)-degrading strains were: *Marinobacter xestospongiae* strain UST090418-1611, *Microbulbifer celer* strain ISL-39, *Shewanella waksmanii* strain KMM 3823, *Tumebacillus lipolyticus* strain NIO-S10, and *Vibrio harveyi* strain NBRC 15634 (S7 Table).

The occurrence of P(3HB)-degrading bacteria was observed in all samples, with the highest to lowest order being: Gram-positive rods of *Tumebacillus* sp. (35%) > Gram-negative rods of *Marinobacter* sp. (23%) > Gram-negative rods of *Shewanella* sp. (19%) > Gram-negative rods of *Microbulbifer* sp. (12%) = Gram-negative rods of *Vibrio* sp. (12%) (Table 3). Bacteria found in both negative and positive controls, as well as P(3HB)-coated concrete, were *Marinobacter* sp., *Microbulbifer* sp., and *Tumebacillus* sp.; *Shewanella* sp. was present in both seawater and P(3HB)-coated concrete, while *Vibrio* sp. was exclusively found on P(3HB)-coated concrete. Notably, all strains were present on P(3HB)-coated concrete, irrespective of the concentration and coating cycles used.

**Fig 8:**
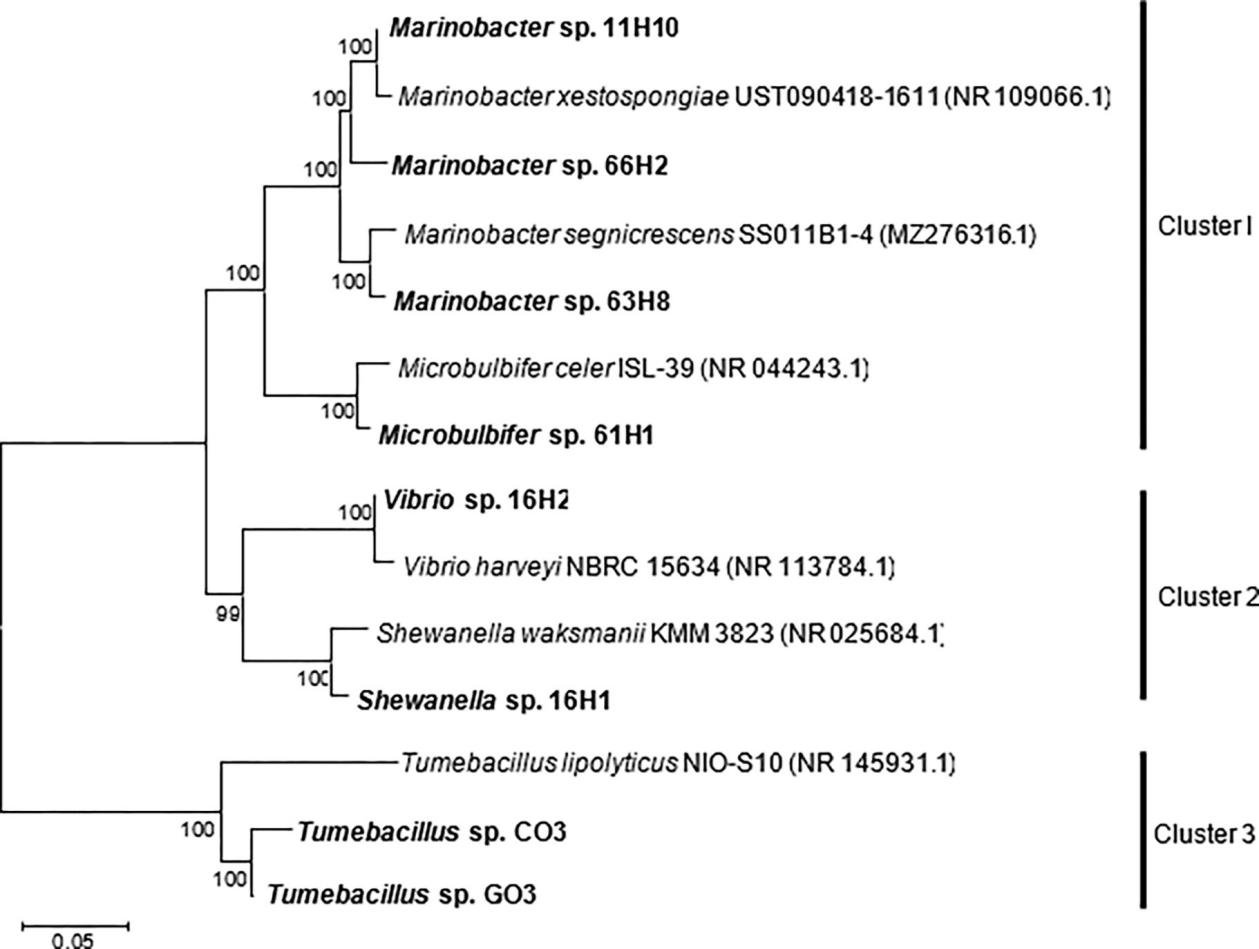
Phylogenetic tree based on 16S rRNA gene sequences comparison among isolated marine P(3HB)-degrading bacteria. The scale bar represents 2% estimated sequence divergence. Bootstrap values (%) were calculated from 1000 trials. Accession numbers are indicated in parentheses.

The phylogenetic analysis of the obtained strains revealed their clustering into four distinct groups with robust bootstrap support (Fig 8). Cluster 1 exhibited a complex structure, consisting of two subclusters. The first subcluster contained strains belonging to the genus *Marinobacter*, while the second subcluster consisted of strains from the genus *Microbulbifer*. Both of these subclusters were affiliated with the family Alteromonadaceae. Cluster 2 consisted of Gram-negative bacteria belonging to the families Shewanellaceae and Vibrionaceae. In contrast, cluster 3 was comprised of Gram-positive bacteria from the family Alicyclobacillaceae, primarily consists of *Tumebacillus* sp.

## Discussion

### Characteristics of P(3HB) coating on concrete

The inherent biodegradability of P(3HB) suggests its potential to enhance the attachment and growth of marine biofilms [42,60]. The distinct peaks observed in the FTIR spectra of all coated samples provide evidence of the successful incorporation of P(3HB) onto the concrete surface, irrespective of the concentration or number of coating cycles applied. The EDX observation made suggests that higher concentrations of P(3HB) result in a more substantial deposition of the coating material on the surface, whereas additional coating cycles do not notably increase material quantity. This observation suggests a saturation threshold in the effective deposition of P(3HB) on concrete surfaces using dip-coating techniques. Conversely, lower concentrations of P(3HB) in a single coating cycle deposit a limited amount of material, potentially resulting in an uneven or insufficient coating layer. To ensure an adequate coating layer, a higher number of coating cycles is required for lower concentrations. Consequently, the relationship between coating cycles and the distribution of P(3HB) material is more pronounced at lower concentrations, necessitating a higher number of cycles for sufficient deposition, whereas at higher concentrations, additional cycles primarily contribute to an even distribution without significantly altering the overall quantity of the coating material (Fig 4, Table 2). In this study, a simple dip-coating method was used but other methods such as spray coating probably would result in different surface properties.

### Biofilm growth patterns on concrete surface

All treatments showed biofilm attachment and growth, except for the negative control (Portland cement concrete, CO). The absence of visible biofilm and low bacterial abundance in the negative control suggest that the lack of specific treatment or surface modification could result in limited biofilm attachment and growth on the concrete surface. Conventional concrete surfaces are highly alkaline, with a pH of up to 13.8 [61], which can severely affect the adhesion and attachment of organisms. Important pioneer species, such as algae, have the best growth rate between pH 7.5 and 8.0 [62–64]. Although this alkaline effect can wear off over time, it takes at least six months of immersion in seawater for the pH value to decrease to the pH of the seawater [65].

In contrast, the positive control concrete (GO), which incorporated seashells, exhibited a bacterial community comparable to that of seawater and P(3HB)-coated concrete. The presence of seashells in the green concrete contributed to its increased surface roughness and micro-scale porous complexity, creating favourable conditions for biofilm formation [66–68]. The higher surface area and irregularities on the GO samples provided protection, facilitated the attachment and growth of biofilm-forming microorganisms, leading to a visually prominent biofilm coverage [69,70]. However, the high standard deviation between bacterial abundance counts of GO replicates suggested that the advantages of complexity-induced biofilm formation may not be consistently stable. This variability could be influenced by various physical and biological conditions, including species-specific responses, as different species may react differently to the induced complexity [71,72].

In the case of P(3HB)-coated concrete, biofilm formation was visibly evident across all treatments, showcasing a more diverse microbial composition and higher abundance compared to both control concretes, as indicated by the distinct colour and bacterial counts (Figs 5 and 6). The colour intensity of the biofilm can serve as a basic yet effective quantitative method for assessing its development, as it is influenced by the presence of pigments like chlorophyll a, carotenoids, and phycobiliproteins [73,74]. While exact bacterial counts in a laboratory setting is impossible, the retrieval of particle-associated bacteria, with a cultivability as high as 25% of the population, can be achieved using the well-established Zobell Marine agar [75]. This ensures a comprehensive coverage of cultivable genera, providing a confident and representative overview of biofilm improvement through P(3HB) coating. The inherent properties of the P(3HB) coating, including its surface characteristics and chemical composition, play a vital role in promoting accelerated biofilm development [76,77].

**Fig 9:**
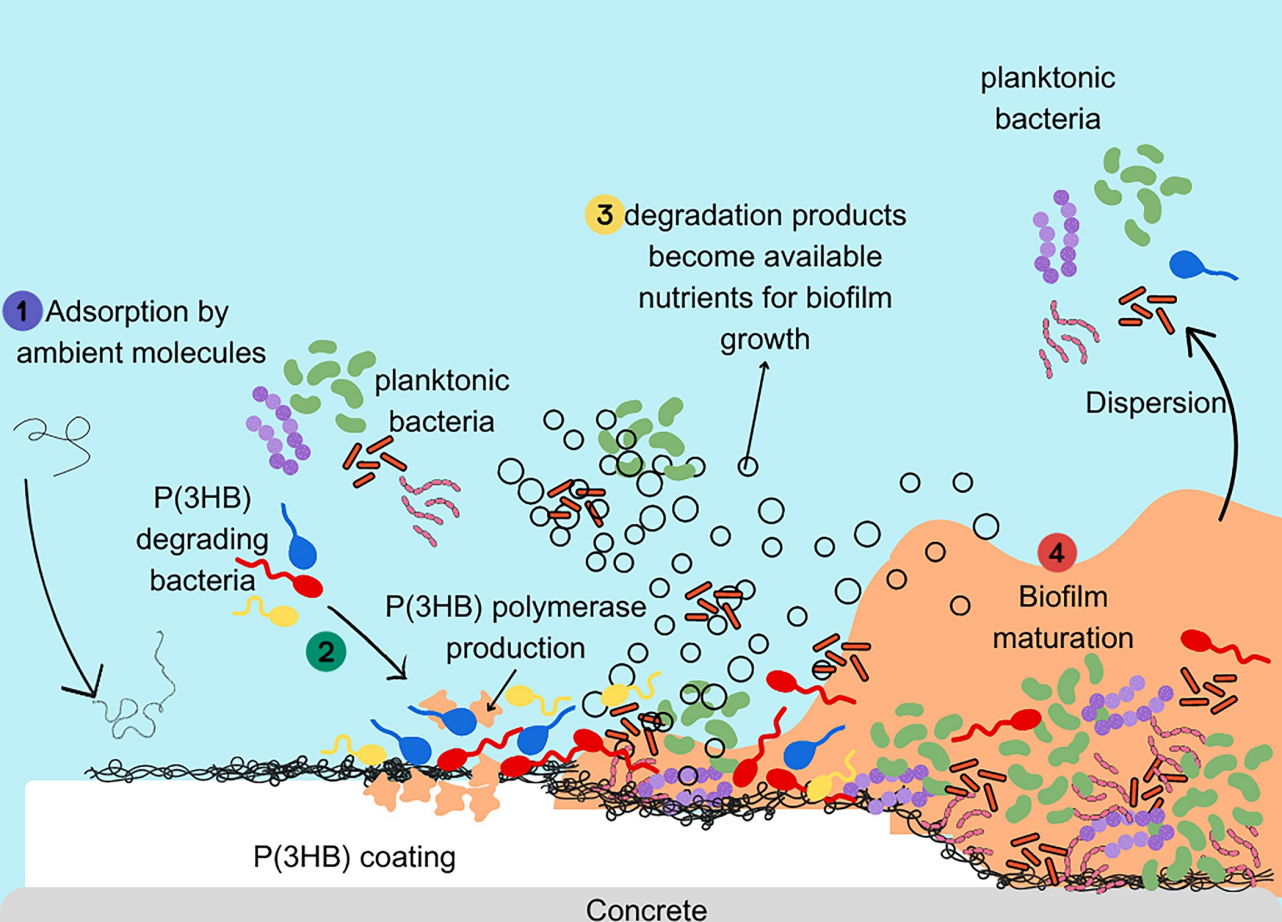
Schematic diagram of degradation of P(3HB)-coated concrete in marine environment.

Different from previous studies that primarily used coatings to increase surface area for biofilm attachment [30,31], the P(3HB) coating functions differently by attracting bacterial communities. It achieves this by regulating the availability of limiting nutrients in the environment [78,79]. P(3HB) is known to be degraded by specific bacteria possessing P(3HB) degradation enzymes. These bacteria, referred to as P(3HB)-degrading bacteria, are capable of utilizing P(3HB) as a carbon source [80]. The presence of P(3HB) as a carbon source on the concrete surface attracts P(3HB)-degrading bacteria, which hydrolyse the P(3HB) into smaller molecules like 3-hydroxybutyric acid and crotonic acid [81]. These breakdown products become nutrients for the metabolic activity of other bacteria, promoting the colonization and biofilm development of both P(3HB)-degrading and non-degrading bacteria [43,82]. The biodegradation process of P(3HB), which includes biodeterioration, biofragmentation, and assimilation, provides additional nutrient sources available in the environment [83,84]. This abundance of nutrients reduces competition among microorganisms and ultimately enhances the bioreceptivity of the coated concrete, promoting the growth and development of diverse microbial communities (Fig 9).

Nevertheless, the single coating layer of P(3HB) at different concentrations (G-11 and G-61) demonstrated similar biofilm coverage to that of the negative control (CO), despite slightly higher bacterial counts (Figs 5 and 6). This observation suggests that the P(3HB) coating, at a single layer, may not have provided enough material or thickness to significantly enhance biofilm formation compared to the negative control. Although increasing either the concentration or coating cycle would substantially improve bacteria count, it is noteworthy that the G-66 treatment, with the highest concentration and coating cycle, exhibited the least biofilm coverage among all P(3HB)-coated concrete samples, even in comparison to the positive control. This discrepancy indicates that the concentration and coating cycles of P(3HB) could have complex and contrasting effects on biofilm development.

The lower biofilm recruitment observed in the G-66 sample could be attributed to several factors. The higher concentration of P(3HB) used in combination with a longer coating cycle resulted in the formation of a thicker and denser coating layer on the concrete surface. This denser coating may have had a selective effect on the types of bacteria that could attach and form biofilms. Non-P(3HB)-degrading bacteria may not have been able to penetrate or adhere effectively to the underlying concrete surface due to the denser coating, thus limiting their access and resulting in lower biofilm coverage. On the other hand, the thicker P(3HB) coating in the G-66 sample created a favourable environment for the growth and colonization of P(3HB)-degrading bacteria, giving them a competitive advantage over non-P(3HB)-degrading bacteria in the biofilm community [79]. This selective advantage led to a dominance of P(3HB)-degrading bacteria in the microbial community of the G-66 sample. However, it is important to note that this shift in the microbial community structure is likely a temporary and influenced by the specific conditions and composition of the coating. Over temporal degradation of the P(3HB) coating and subsequent nutrient release, a probable alteration in the microbial community composition could transpire, encompassing a more diverse array of bacterial species [85]. This notion was supported by the exchange of concrete samples on which CFU counts were performed, revealing G-66 to manifest the highest CFU abundance with minimal variance. This observation implies a substantial bacterial population attracted to G-66, although their dispersion might be constrained spatially or physiochemically.

Additionally, different bacterial species have varying capabilities to form biofilms based on their genetic makeup and environmental factors. For example, some bacteria like *Pseudomonas aeruginosa* are known to form biofilms in various conditions [86], while strains of *Escherichia coli* may only form biofilms under nutrient-limited conditions [87]. In the case of the G-66 sample, the high concentration of degraded P(3HB) coating may have provided an abundant nutrient source for bacteria, resulting in a high nutrient condition. In such conditions, certain bacteria may prioritize planktonic growth and utilize the readily available nutrients without forming a visible biofilm structure [88]. Another possible scenario is that the high concentration of P(3HB) used in the study may have exhibited an antiadhesive effect against bacteria, leading to a reduction in biofilm formation. Previous studies have reported the antiadhesive activity of P(3HB) against *Vibrios*, with inhibition rates exceeding 80% [89]. This antiadhesive effect can interfere with the initial attachment and colonization of bacteria on the G-66 sample surface, thereby reducing biofilm formation. This antiadhesive effect does not necessarily inhibit the growth of bacteria, as evidenced by the highest number of bacteria count in the G-66 sample.

### Microbial P(3HB) degraders

In natural marine conditions, there is an abundance of bacteria capable of degrading polyhydroxyalkanoates, widely distributed in various environments, including marine sediment, seawater, and the plastisphere of various materials [37,83]. However, the efficiency of biodegradation by microorganisms is influenced by the specific type of bioplastic and the environment it is exposed to [90]. To assess the degree of bioreceptivity induced by P(3HB) coating on concrete in the marine environment, it is important to identify the P(3HB)-degrading bacteria. This information is crucial for estimating the biodegradation behaviour of P(3HB)-coated concrete in the marine environment and understanding its potential environmental impact.

Among the isolated bacteria, representatives of the genera *Vibrio*, *Shewanella*, *Marinobacter*, and *Microbulbifer* have been previously reported as marine PHA-degrading bacteria in the literature [89,91–94]. However, there is a notable absence of literature reporting the PHA depolymerase activity specifically from representatives of the genus *Tumebacillus*. Belonging to the family Alicyclobacillaceae within the order Bacillales, *Tumebacillus* species have been isolated from various environments, such as soil, river water, rhizosphere, and algal scum [95–99]. This study marks the first instance of isolating a strain from the genus *Tumebacillus* in the marine environment. While nine known species exist in this genus, only seven strains have undergone whole-genome sequencing. Among these, *Tumebacillus amylolyticus* ITR2 (accession number: GCA_016722965.1), *Tumebacillus permanentifrigoris* DSM 18773 (GCA_003148565.1) and *Tumebacillus* sp. DT12 (GCA_026410385.1) possess putative extracellular PHA depolymerase genes [100–102]. Despite the presence of these putative genes, no *Tumebacillus* sp. strains have been previously reported to degrade P(3HB). It is imperative to underscore that our study highlights the identification of a marine *Tumebacillus* sp. strain with the inherent capability of degrading P(3HB). However, it is crucial to clarify that our testing methodology involves evaluating a microbial population inclusive of the *Tumebacillus* sp. strain. Further analyses are warranted to comprehensively assess the species’ capabilities and properties in this context.

The presence and abundance of P(3HB)-degrading bacteria did not show a clear correlation with the P(3HB) concentration or coating cycles in our study. P(3HB)-degrading bacteria were found across all treatment surfaces and controls, with no significant difference observed between them (Table 3). While the P(3HB) concentration and coating cycles do play a role in providing sufficient substrate for bacterial growth and activity, they alone do not dictate the population of P(3HB)-degrading bacteria. The higher P(3HB) concentration did not necessary attract more P(3HB)-degrading bacteria. It is important to exercise caution when interpreting these results, as the isolation process using different agar media may have influenced the growth of bacterial communities, and thus, the isolated strains may not fully represent the entire community and its degradation ability.

The manipulation of P(3HB) concentration and coating cycle in current study revealed that these factors play little role in attracting P(3HB)-degrading bacteria but contribute to promoting biofilm development. Microbial communities consist of diverse populations of microorganisms with distinct metabolic capabilities and preferences. These variations can influence their ability to utilize P(3HB) as a carbon source, form biofilms, and degrade the polymer [103]. While P(3HB) concentration and coating cycle can provide the necessary substrate and physical environment, it is the composition and dynamics of the microbial communities that ultimately dictate the efficiency and extent of biofilm formation and P(3HB) degradation [104].

In the present study, the degradation rate of P(3HB) coatings in the actual marine environment was not measured, and the exact timeframe for complete degradation remains unknown. While the presence of the coatings initially facilitated accelerated biofilm formation, it is possible that once the coatings are fully degraded, the biofilm development may return to the typical levels observed on uncoated surfaces. The degradation of the coatings leads to the loss of specific surface properties or factors that initially enhanced biofilm formation, such as the supplies of degradation products. Consequently, the underlying substrata surface morphology and other enhancing or limiting factors become crucial in determining biofilm development. Furthermore, in the current study, we did not examine the microbial populations on the P(3HB) coatings. Understanding the microbial community developed on the coating and its potential effects on ecological succession would provide valuable insights. The microbial community composition and dynamics on the coating could influence the subsequent stages of biofilm development and the overall microbial ecology in the system.

To comprehensively evaluate the long-term effects of P(3HB) coatings on biofilm development, it is crucial to investigate the degradation kinetics of the coatings, monitor the biofilm development even after complete degradation of the coatings, and analyze the microbial communities associated with the coatings throughout the degradation process. These investigations would offer valuable insights into the temporal dynamics of biofilm formation and microbial succession on the coatings.

To comprehend the ecological implications of utilizing P(3HB) coatings in ecological engineering applications, such as greening the grey, a holistic assessment of how the coatings influence biofilm development and microbial communities over time is essential. This knowledge will be instrumental in evaluating the overall performance, durability, and environmental impacts of P(3HB) coatings in ecological engineering endeavours. By understanding the interactions between P(3HB) coatings and microbial communities, we can optimize their design and application to promote sustainable and eco-friendly solutions in various environmental contexts.

This study presents novel findings on the degradation of P(3HB) coatings in the marine environment and its potential to promote microbial succession on concrete surfaces. Our results clearly demonstrate that the addition of P(3HB) coatings onto concrete surfaces leads to accelerated biofilm formation, enhancing the bioreceptivity of the concrete. Interestingly, Our observations indicate that biofilm formation was not solely influenced by the concentration of P(3HB). This suggests that even at lower concentrations, increasing the number of coating cycles may effectively promote biofilm development.

Based on our findings, we recommend using a 1% or 6% w/v concentration of P(3HB) with three coating cycles for optimal results. This combination ensures a homogeneous layer that maximizes the assimilation of the concrete surface for biofilm colonization. Further increases in the coating cycles did not result in a significant improvement in biofilm formation, indicating that optimal results can be achieved with this specific application protocol.

In conclusion, this study provides valuable knowledge on the use of P(3HB) coatings to enhance the bioreceptivity of concrete surfaces and promote biofilm development. The findings contribute to the advancement of ecological engineering and provide a foundation for further research and application of P(3HB) coatings in various environmental and biotechnological contexts.

## Supporting information

S1 Fig Clear zone formation due to depolymerase activity of P(3HB)-degrading bacteria. Treatment (A) seawater, (B) CO, (C) GO, (D) G-11, (E) G-13, (F) G-16, (G) G-61, (H) G-63 and (I) G-66.
(DOCX)

S1 Table Sample mix designs were analyzed for their mechanical properties at the age of 28 days. The mechanical properties were presented as the mean ± standard deviation.
(DOCX)

S2 Table Descriptive statistics of one-way ANOVA for coating cycles on deposited carbon quantity. (DOCX)

S3 Table Descriptive statistics of two-way ANOVA for concentration and coating cycles on bacterial abundance. (DOCX)

S4 Table Descriptive statistics of Tukey’s Honest Significant Difference test for concentration and coating cycles on bacterial abundance. (DOCX)

S5 Table The CFU counts recorded for all treatments at 72 hours. Averages along with standard deviations were calculated based on triplicate measurements.
(DOCX)

S6 Table Descriptive statistics of two-way ANOVA for concentration and coating cycles on isolated P(3HB) degraders. (DOCX)

S7 Table Isolated marine P(3HB)-degrading bacteria. (DOCX)
