# Supplementary material for: Effects of poly(3-hydroxybutyrate) [P(3HB)] coating on the bacterial communities of artificial structures
Source: PLoS One. 2024 Apr 18;19(4):e0300929. doi: 10.1371/journal.pone.0300929 (PMC11025745; doi:10.1371/journal.pone.0300929)
Supplement: S1 Table — The mechanical properties were presented as the mean ± standard deviation. (DOCX) [file pone.0300929.s002.docx]

Effects of poly(3-hydroxybutyrate) [P(3HB)] coating on the bacterial communities of artificial structures

Yee Jean Chai^1^, Taufiq Ahmad Syauqi^2^, Kumar Sudesh^2^, Tan Leng Ee^3,#a^, Cheah Chee Ban^3^, Amanda Chong Kar Mun^1^, Elisabeth Marijke Anne Strain^4,5^, Faradina Merican^2^, Masazurah A. Rahim^6^, Kaharudin Md Salleh^6^, Chee Su Yin^1^*

^1^Centre for Global Sustainability Studies, Universiti Sains Malaysia, Minden, Penang, Malaysia

^2^School of Biological Sciences, Universiti Sains Malaysia, Minden, Penang, Malaysia

^3^School of Housing, Building and Planning, Universiti Sains Malaysia, Minden, Penang, Malaysia

^4^Institute for Marine and Antarctic Studies, University of Tasmania, Hobart, Australia

^5^Centre for Marine Socioecology, University of Tasmania, Hobart, Australia

^6^Fisheries Research Institute, Batu Maung, Penang, Malaysia

^#a^Current Address: Faculty of Built Environment, Department of Construction Management, Tunku Abdul Rahman University of Management and Technology, Setapak, Kuala Lumpur, Malaysia

*Corresponding author

E-mail: suyinchee@usm.my (CSY)

# **Supporting information**

**S1 Table. Sample mix designs were analyzed for their mechanical properties at the age of 28 days. The mechanical properties were presented as the mean ± standard deviation.**

| Mix design | Portland cement concrete | Green concrete |
| --- | --- | --- |
| Cement (kg/m^3^) | 630 | 315 |
| GGBS (kg/m^3^) | 0 | 315 |
| Sand (kg/m^3^) | 1418 | 0 |
| Quarry dust (kg/m^3^) | 0 | 709 |
| Seashells (kg/m^3^) | 0 | 709 |
| Water to binder ratio | 0.4 | 0.4 |
| Flow (mm) | 140 | 140 |
| Fineness modulus | 2.6 | 2.6 |
| Compressive strength (MPa) | 68.73 ± 10.66 | 78.77 ± 9.25 |
| Flexural strength (MPa) | 11.97 ± 0.79 | 12.36 ± 0.66 |
| Water absorption (%) | 1.08 ± 0.04 | 0.63 ± 0.09 |
| Porosity (%) | 7.69 ± 0.16 | 5.77 ± 0.36 |
