# Supplementary material for: Effects of poly(3-hydroxybutyrate) [P(3HB)] coating on the bacterial communities of artificial structures
Source: PLoS One. 2024 Apr 18;19(4):e0300929. doi: 10.1371/journal.pone.0300929 (PMC11025745; doi:10.1371/journal.pone.0300929)
Supplement: S3 Table — (DOCX) [file pone.0300929.s004.docx]

Effects of poly(3-hydroxybutyrate) [P(3HB)] coating on the bacterial communities of artificial structures

Yee Jean Chai^1^, Taufiq Ahmad Syauqi^2^, Kumar Sudesh^2^, Tan Leng Ee^3,#a^, Cheah Chee Ban^3^, Amanda Chong Kar Mun^1^, Elisabeth Marijke Anne Strain^4,5^, Faradina Merican^2^, Masazurah A. Rahim^6^, Kaharudin Md Salleh^6^, Chee Su Yin^1^*

^1^Centre for Global Sustainability Studies, Universiti Sains Malaysia, Minden, Penang, Malaysia

^2^School of Biological Sciences, Universiti Sains Malaysia, Minden, Penang, Malaysia

^3^School of Housing, Building and Planning, Universiti Sains Malaysia, Minden, Penang, Malaysia

^4^Institute for Marine and Antarctic Studies, University of Tasmania, Hobart, Australia

^5^Centre for Marine Socioecology, University of Tasmania, Hobart, Australia

^6^Fisheries Research Institute, Batu Maung, Penang, Malaysia

^#a^Current Address: Faculty of Built Environment, Department of Construction Management, Tunku Abdul Rahman University of Management and Technology, Setapak, Kuala Lumpur, Malaysia

*Corresponding author

E-mail: suyinchee@usm.my (CSY)

# **Supporting information**

**S3 Table. Descriptive statistics of two-way ANOVA for concentration and coating cycles on bacterial abundance.**

call: aov(formula = count ~ conc + cycle + conc:cycle, data = cfu)

Df Sum Sq Mean Sq F value Pr(>F)

conc 2 233497029 116748514 5.098 0.01625 *

cycle 2 359110561 179555280 7.840 0.00306 **

conc:cycle 2 265210676 132605338 5.790 0.01038 *

Residuals 20 458030248 22901512

Signif. codes: 0 ‘***’ 0.001 ‘**’ 0.01 ‘*’ 0.05 ‘.’ 0.1 ‘ ’ 1
