# Supplementary material for: Effects of poly(3-hydroxybutyrate) [P(3HB)] coating on the bacterial communities of artificial structures
Source: PLoS One. 2024 Apr 18;19(4):e0300929. doi: 10.1371/journal.pone.0300929 (PMC11025745; doi:10.1371/journal.pone.0300929)
Supplement: S4 Table — (DOCX) [file pone.0300929.s005.docx]

Effects of poly(3-hydroxybutyrate) [P(3HB)] coating on the bacterial communities of artificial structures

Yee Jean Chai^1^, Taufiq Ahmad Syauqi^2^, Kumar Sudesh^2^, Tan Leng Ee^3,#a^, Cheah Chee Ban^3^, Amanda Chong Kar Mun^1^, Elisabeth Marijke Anne Strain^4,5^, Faradina Merican^2^, Masazurah A. Rahim^6^, Kaharudin Md Salleh^6^, Chee Su Yin^1^*

^1^Centre for Global Sustainability Studies, Universiti Sains Malaysia, Minden, Penang, Malaysia

^2^School of Biological Sciences, Universiti Sains Malaysia, Minden, Penang, Malaysia

^3^School of Housing, Building and Planning, Universiti Sains Malaysia, Minden, Penang, Malaysia

^4^Institute for Marine and Antarctic Studies, University of Tasmania, Hobart, Australia

^5^Centre for Marine Socioecology, University of Tasmania, Hobart, Australia

^6^Fisheries Research Institute, Batu Maung, Penang, Malaysia

^#a^Current Address: Faculty of Built Environment, Department of Construction Management, Tunku Abdul Rahman University of Management and Technology, Setapak, Kuala Lumpur, Malaysia

*Corresponding author

E-mail: suyinchee@usm.my (CSY)

# **Supporting information**

**S4 Table. Descriptive statistics of Tukey’s Honest Significant Difference test for concentration and coating cycles on bacterial abundance.**

Tukey multiple comparisons of means

95% family-wise confidence level

Fit: aov(formula = count ~ conc + cycle + conc * cycle, data = cfu)

$conc

diff lwr upr p adj

six-one 7162.496 1455.031 12869.961 0.0126413

zero-one 2917.803 -2789.662 8625.267 0.4150844

zero-six -4244.693 -9952.158 1462.771 0.1699468

$cycle

diff lwr upr p adj

six-one 10814.9352 3081.643 18548.2278 0.0043916

three-one 3973.7580 -3759.535 11707.0507 0.4914120

zero-one 4929.5644 -2129.934 11989.0624 0.2380982

three-six -6841.1772 -14574.470 892.1155 0.0947455

zero-six -5885.3708 -12944.869 1174.1273 0.1237802

zero-three 955.8064 -6103.692 8015.3044 0.9809365

$`conc:cycle`

diff lwr upr p adj

six:one-one:one 670.1803 -13694.280 15034.6412 1.0000000

one:six-one:one 2177.7103 -12186.750 16542.1712 0.9999818

six:six-one:one 20122.3403 5757.880 34486.8012 0.0021666

one:three-one:one 2872.5093 -11491.951 17236.9702 0.9997296

six:three-one:one 5745.1870 -8619.274 20109.6478 0.9325307

zero:zero-one:one 4601.2092 -7127.324 16329.7424 0.9400701

one:six-six:one 1507.5300 -12856.931 15871.9908 0.9999996

six:six-six:one 19452.1600 5087.699 33816.6208 0.0031442

one:three-six:one 2202.3290 -12162.132 16566.7898 0.9999796

six:three-six:one 5075.0067 -9289.454 19439.4675 0.9697893

zero:zero-six:one 3931.0289 -7797.504 15659.5620 0.9791401

six:six-one:six 17944.6300 3580.169 32309.0908 0.0072579

one:three-one:six 694.7990 -13669.662 15059.2598 1.0000000

six:three-one:six 3567.4767 -10796.984 17931.9375 0.9980822

zero:zero-one:six 2423.4989 -9305.034 14152.0320 0.9996327

one:three-six:six -17249.8310 -31614.292 -2885.3702 0.0106474

six:three-six:six -14377.1533 -28741.614 -12.6925 0.0496734

zero:zero-six:six -15521.1311 -27249.664 -3792.5980 0.0040212

six:three-one:three 2872.6777 -11491.783 17237.1385 0.9997294

zero:zero-one:three 1728.6999 -9999.833 13457.2330 0.9999863

zero:zero-six:three -1143.9778 -12872.511 10584.5554 0.9999998
