# Supplementary material for: Effects of poly(3-hydroxybutyrate) [P(3HB)] coating on the bacterial communities of artificial structures
Source: PLoS One. 2024 Apr 18;19(4):e0300929. doi: 10.1371/journal.pone.0300929 (PMC11025745; doi:10.1371/journal.pone.0300929)
Supplement: S5 Table — Averages along with standard deviations were calculated based on triplicate measurements. (DOCX) [file pone.0300929.s006.docx]

Effects of poly(3-hydroxybutyrate) [P(3HB)] coating on the bacterial communities of artificial structures

Yee Jean Chai^1^, Taufiq Ahmad Syauqi^2^, Kumar Sudesh^2^, Tan Leng Ee^3,#a^, Cheah Chee Ban^3^, Amanda Chong Kar Mun^1^, Elisabeth Marijke Anne Strain^4,5^, Faradina Merican^2^, Masazurah A. Rahim^6^, Kaharudin Md Salleh^6^, Chee Su Yin^1^*

^1^Centre for Global Sustainability Studies, Universiti Sains Malaysia, Minden, Penang, Malaysia

^2^School of Biological Sciences, Universiti Sains Malaysia, Minden, Penang, Malaysia

^3^School of Housing, Building and Planning, Universiti Sains Malaysia, Minden, Penang, Malaysia

^4^Institute for Marine and Antarctic Studies, University of Tasmania, Hobart, Australia

^5^Centre for Marine Socioecology, University of Tasmania, Hobart, Australia

^6^Fisheries Research Institute, Batu Maung, Penang, Malaysia

^#a^Current Address: Faculty of Built Environment, Department of Construction Management, Tunku Abdul Rahman University of Management and Technology, Setapak, Kuala Lumpur, Malaysia

*Corresponding author

E-mail: suyinchee@usm.my (CSY)

# **Supporting information**

**S5 Table. The CFU counts recorded for all treatments at 72 hours.**Averages along with standard deviations were calculated based on triplicate measurements.

| Treatment | Seawater | CO | GO | G-11 | G-13 | G-16 | G-61 | G-63 | G-66 |
| --- | --- | --- | --- | --- | --- | --- | --- | --- | --- |
| CFU/ml | 1.1 × 10^4^ | 3.9 × 10^3^ | 2.3 × 10^4^ | 4.5 × 10^3^ | 7.6 × 10^3^ | 6.3 × 10^3^ | 4.7 × 10^3^ | 9.5 × 10^3^ | 2.3 × 10^4^ |
| Standard deviation | 2.5 × 10^3^ | 6.5 × 10^2^ | 1.7 × 10^4^ | 1.8 × 10^3^ | 4.1 × 10^3^ | 3.0 × 10^3^ | 1.9 × 10^3^ | 5.1 × 10^3^ | 7.0 × 10^3^ |
