# Supplementary material for: Effects of poly(3-hydroxybutyrate) [P(3HB)] coating on the bacterial communities of artificial structures
Source: PLoS One. 2024 Apr 18;19(4):e0300929. doi: 10.1371/journal.pone.0300929 (PMC11025745; doi:10.1371/journal.pone.0300929)
Supplement: S6 Table — (DOCX) [file pone.0300929.s007.docx]

Effects of poly(3-hydroxybutyrate) [P(3HB)] coating on the bacterial communities of artificial structures

Yee Jean Chai^1^, Taufiq Ahmad Syauqi^2^, Kumar Sudesh^2^, Tan Leng Ee^3,#a^, Cheah Chee Ban^3^, Amanda Chong Kar Mun^1^, Elisabeth Marijke Anne Strain^4,5^, Faradina Merican^2^, Masazurah A. Rahim^6^, Kaharudin Md Salleh^6^, Chee Su Yin^1^*

^1^Centre for Global Sustainability Studies, Universiti Sains Malaysia, Minden, Penang, Malaysia

^2^School of Biological Sciences, Universiti Sains Malaysia, Minden, Penang, Malaysia

^3^School of Housing, Building and Planning, Universiti Sains Malaysia, Minden, Penang, Malaysia

^4^Institute for Marine and Antarctic Studies, University of Tasmania, Hobart, Australia

^5^Centre for Marine Socioecology, University of Tasmania, Hobart, Australia

^6^Fisheries Research Institute, Batu Maung, Penang, Malaysia

^#a^Current Address: Faculty of Built Environment, Department of Construction Management, Tunku Abdul Rahman University of Management and Technology, Setapak, Kuala Lumpur, Malaysia

*Corresponding author

E-mail: suyinchee@usm.my (CSY)

# **Supporting information**

**S6 Table. Descriptive statistics of two-way ANOVA for concentration and coating cycles on isolated P(3HB) degraders.**

call: aov(formula = isolate ~ conc + cycle + conc:cycle, data = iso)

Df Sum Sq Mean Sq F value Pr(>F)

cycle 3 9.375 3.1250 6.250 0.284

conc 1 0.667 0.6667 1.333 0.454

cycle:conc 2 0.333 0.1667 0.333 0.775

Residuals 1 0.500 0.5000

Signif. codes: 0 ‘***’ 0.001 ‘**’ 0.01 ‘*’ 0.05 ‘.’ 0.1 ‘ ’ 1
