# Supplementary material for: Effects of poly(3-hydroxybutyrate) [P(3HB)] coating on the bacterial communities of artificial structures
Source: PLoS One. 2024 Apr 18;19(4):e0300929. doi: 10.1371/journal.pone.0300929 (PMC11025745; doi:10.1371/journal.pone.0300929)
Supplement: S7 Table — (DOCX) [file pone.0300929.s008.docx]

Effects of poly(3-hydroxybutyrate) [P(3HB)] coating on the bacterial communities of artificial structures

Yee Jean Chai^1^, Taufiq Ahmad Syauqi^2^, Kumar Sudesh^2^, Tan Leng Ee^3,#a^, Cheah Chee Ban^3^, Amanda Chong Kar Mun^1^, Elisabeth Marijke Anne Strain^4,5^, Faradina Merican^2^, Masazurah A. Rahim^6^, Kaharudin Md Salleh^6^, Chee Su Yin^1^*

^1^Centre for Global Sustainability Studies, Universiti Sains Malaysia, Minden, Penang, Malaysia

^2^School of Biological Sciences, Universiti Sains Malaysia, Minden, Penang, Malaysia

^3^School of Housing, Building and Planning, Universiti Sains Malaysia, Minden, Penang, Malaysia

^4^Institute for Marine and Antarctic Studies, University of Tasmania, Hobart, Australia

^5^Centre for Marine Socioecology, University of Tasmania, Hobart, Australia

^6^Fisheries Research Institute, Batu Maung, Penang, Malaysia

^#a^Current Address: Faculty of Built Environment, Department of Construction Management, Tunku Abdul Rahman University of Management and Technology, Setapak, Kuala Lumpur, Malaysia

*Corresponding author

E-mail: suyinchee@usm.my (CSY)

# **Supporting information**

**S7 Table. Isolated marine P(3HB)-degrading bacteria.**

| Isolate | Related strains | Query cover (%) | Percent identity (%) |
| --- | --- | --- | --- |
| 66H2 | *Marinobacter xestospongiae* strain UST090418-1611 | 99 | 97.93 |
| 61H1 | *Microbulbifer celer* strain ISL-39 | 100 | 99.22 |
| 16H1 | *Shewanella waksmanii* strain KMM 3823 | 99 | 98.59 |
| CO3 | *Tumebacillus lipolyticus* strain NIO-S10 | 99 | 94.06 |
| 11H8 | *Vibrio harveyi* strain NBRC 15634 | 99 | 99.86 |
